# Whole-Genome Sequencing for Tracing the Genetic Diversity of *Brucella abortus* and *Brucella melitensis* Isolated from Livestock in Egypt

**DOI:** 10.3390/pathogens10060759

**Published:** 2021-06-16

**Authors:** Aman Ullah Khan, Falk Melzer, Ashraf E. Sayour, Waleed S. Shell, Jörg Linde, Mostafa Abdel-Glil, Sherif A. G. E. El-Soally, Mandy C. Elschner, Hossam E. M. Sayour, Eman Shawkat Ramadan, Shereen Aziz Mohamed, Ashraf Hendam, Rania I. Ismail, Lubna F. Farahat, Uwe Roesler, Heinrich Neubauer, Hosny El-Adawy

**Affiliations:** 1 Institute of Bacterial Infections and Zoonoses, Friedrich-Loeffler-Institut, 07743 Jena, Germany; AmanUllah.Khan@fli.de (A.U.K.); falk.melzer@fli.de (F.M.); Joerg.Linde@fli.de (J.L.); Mostafa.AbdelGlil@fli.de (M.A.-G.); mandy.elschner@fli.de (M.C.E.); Heinrich.neubauer@fli.de (H.N.); 2 Institute for Animal Hygiene and Environmental Health, Free University of Berlin, 14163 Berlin, Germany; uwe.roesler@fu-berlin.de; 3 Department of Pathobiology, University of Veterinary and Animal Sciences (Jhang Campus), Lahore 54000, Pakistan; 4 Department of Brucellosis, Animal Health Research Institute, Agricultural Research Center, Dokki, Giza 12618, Egypt; sayourashraf@gmail.com (A.E.S.); raniaismail22@yahoo.com (R.I.I.); 5 Central Laboratory for Evaluation of Veterinary Biologics, Agricultural Research Center, Abbassia, Cairo 11517, Egypt; tarikwaleedshell@hotmail.com; 6 Department of Pathology, Faculty of Veterinary Medicine, Zagazig University, Elzera’a Square, Zagazig 44519, Egypt; 7 Veterinary Service Department, Armed Forces Logistics Authority, Egyptian Armed Forces, Nasr City, Cairo 11765, Egypt; dr.sherifelsoaaly@gmail.com; 8 Biomedical Chemistry Unit, Department of Chemistry and Nutritional Deficiency Disorders, Animal Health Research Institute, Agricultural Research Center, Dokki, Giza 12618, Egypt; Soomy111@yahoo.com; 9 Animal Reproduction Research Institute, Agricultural Research Center, Al Ahram, Giza 12556, Egypt; emanramadan1311971@gmail.com; 10 Veterinary Serum and Vaccine Research Institute, Agricultural Research Center, Abbassia, Cairo 11517, Egypt; shereenibraheem1968@gmail.com (S.A.M.); lubnafady@gmail.com (L.F.F.); 11 Climate Change Information Center, Renewable Energy and Expert Systems (CCICREES), Agricultural Research Center, 9 Algamaa Street, Giza 12619, Egypt; a_hendam@hotmail.com; 12 Faculty of Veterinary Medicine, Kafrelsheikh University, Kafr El-Sheikh 33516, Egypt

**Keywords:** *Brucella*, WGS, SNP, genotyping, epidemiological map, Egypt

## Abstract

Brucellosis is a highly contagious zoonosis that occurs worldwide. Whole-genome sequencing (WGS) has become a widely accepted molecular typing method for outbreak tracing and genomic epidemiology of brucellosis. Twenty-nine *Brucella* spp. (eight *B. abortus* biovar 1 and 21 *B. melitensis* biovar 3) were isolated from lymph nodes, milk, and fetal abomasal contents of infected cattle, buffaloes, sheep, and goats originating from nine districts in Egypt. The isolates were identified by microbiological methods and matrix-assisted laser desorption ionization time-of-flight mass spectrometry (MALDI-TOF MS). Differentiation and genotyping were confirmed using multiplex PCR. Illumina MiSeq^®^ was used to sequence the 29 *Brucella* isolates. Using MLST typing, ST11 and ST1 were identified among *B. melitensis* and *B. abortus*, respectively. *Brucella abortus* and *B. melitensis* isolates were divided into two main clusters (clusters 1 and 2) containing two and nine distinct genotypes by core-genome SNP analysis, respectively. The genotypes were irregularly distributed over time and space in the study area. Both Egyptian *B. abortus* and *B. melitensis* isolates proved to be genomically unique upon comparison with publicly available sequencing from strains of neighboring Mediterranean, African, and Asian countries. The antimicrobial resistance mechanism caused by mutations in rpoB, gyrA, and gyrB genes associated with rifampicin and ciprofloxacin resistance were identified. To the best of our knowledge, this is the first study investigating the epidemiology of *Brucella* isolates from livestock belonging to different localities in Egypt based on whole genome analysis.

## 1. Introduction

Brucellosis is one of the most widespread zoonoses of public health significance and cause of substantial economic losses in animal production systems globally [1,2]. Brucellosis is well controlled in developed countries but is still endemic in the Middle East and Central Asian regions, with high case numbers recorded in humans [3].

The disease is caused by bacteria of the genus *Brucella.* These bacteria are intracellular, Gram-negative, non-motile, and non-spore forming coccobacilli. At present, twelve *Brucella* species are recognized with apparent host preference. However, cross infections of brucellae in unusual animal hosts are also reported [4,5]. *Brucella melitensis* is the most pathogenic species as it is most often isolated from human brucellosis cases and provokes severe disease outcomes [1]. Infection with brucellae results in reproductive disorders in domestic animals and chronic debilitating disease in humans [6].

Brucellosis is endemic in Egypt [7]. Isolation of *B. suis*, *B. melitensis,* and *B. abortus* from livestock has been reported recently [8,9]. *Brucella abortus* and *B. melitensis* have also been isolated from humans, but *B. suis* has thus far only been identified in cattle, pigs, and camels [10,11,12,13]. Like in many other countries of the Mediterranean Basin, *B. melitensis* is the predominant *Brucella* spp. in Egypt causing infections in animals as well as in humans [14].

Various methods are used to identify *Brucella* species and their biovars. Both traditional biochemical and modern molecular typing methods are included in epidemiological investigations. Classical biotyping methods recommended by the World Organization for Animal Health (OIE) are based on phage typing, biochemical, and serological characteristics [15]. These methods are laborious and require the handling of viable bacteria, yet their resolution power is too low for the Egyptian scenario where *B. melitensis* biovar 3 and *B. abortus* biovar 1 are almost exclusively isolated [8,16,17]. The monomorphism of brucellae hinders differentiation at the strain level [18].

The genome of *Brucella* is very stable and is made of two circular chromosomes of approximately 2.1 and 1.2 Mb [19]. *Brucella suis*, *B. melitensis,* and *B. abortus* share more than 90% of their genome [20]. Molecular epidemiology and typing of *Brucella* could be challenging owing to the minor genetic variations in its genome [21]. Pulsed-field gel electrophoresis (PFGE) and amplified fragment length polymorphism (AFLP) typing lack intra- and interlaboratory reproducibility [22]. Therefore, molecular typing methods were developed for evolutionary and epidemiological studies, respectively [1,23,24].

At present, multiple loci variable-number tandem repeat analysis (MLVA) is considered an efficient standardized typing method with good discriminating resolution [25]. However, this typing method has several weaknesses related both to the nature of variable-number tandem repeats (VNTRs) as well as to the laboratory demands of the technique itself [1].

Whole-genome sequencing (WGS) is considered the ideal tool to study genomic variations of organisms in detail. With the advancement and development of next-generation sequencing technologies, complete bacterial genome sequencing has become easy, economical, and highly accessible [26]. Whole-genome sequences provide the most comprehensive collection of genetic variation of an individual, enabling the development of bioinformatic pipelines with optimized resolution for discriminating between even such closely related bacteria as *Brucella* [1,3,26,27,28]. These tools can be tailored for studying outbreaks, geographical distribution, and newly emerging agents [29]. Gene-by-gene comparison using core-genome multilocus sequence typing (cgMLST) and single-nucleotide polymorphism (SNP) calling will replace the current gold standard typing techniques soon [1,26,27]. Recently, MLVA has been used for the epidemiological tracing of *B. melitensis* genotypes in Egypt, comparing them with their related global counterparts [8]. The predominance of *B. melitensis* bv3 throughout the country has compromised the assessment of the epidemiological situation, dictating the use of the latest WGS technology to assist the control of brucellosis in Egypt.

Whole-genome sequencing of brucellae from Mediterranean and African countries revealed the prevalence of *B. melitensis* [27]. The prevalence of *B. melitensis* in Egypt and surrounding countries demands high throughput technology like WGS based sequencing analysis to identify the genetic diversity of brucellae circulating in Egypt and countries in the vicinity.

This study aimed to assess the genetic diversity/relatedness and to trace virulence and antimicrobial resistant (AMR) genes in *B. abortus* and *B. melitensis* strains circulating within Egyptian farm animals through WGS analysis with comparison to neighboring countries.

## 2. Materials and Methods

### 2.1. Brucella Strain Isolation and Identification

Twenty-nine *Brucella* spp. were isolated from clinical specimens of lymph nodes and milk and fetal stomach contents collected from infected cattle, buffaloes, sheep, and goats from Giza, Beheira, Damanhour-Beheira, Asyut, Qalyubia, Beni-Suef, Ismailia, Dakahlia, and Monufia governorates/districts in Egypt (Table S1).

Isolation of *Brucella* spp. was performed according to the OIE standard protocol [15]. Briefly, collected samples were inoculated onto calf blood agar, *Brucella* enriched medium, and *Brucella* selective medium plates (Oxoid GmbH, Wesel, Germany) and incubated at 37 °C in the absence and presence of 5–10% CO_2_ for up to 2 weeks.

The colonies showing typical, round, glistening, pinpoint, and honey drop-like appearance were picked and stained with Gram and modified Ziehl-Neelsen (MZN) staining techniques. The grown colonies were subsequently identified using biochemical tests, motility tests, hemolysis on blood agar, and agglutination with monospecific sera.

Bacterial identification was additionally carried out using MALDI-TOF MS Ultraflex instrument (Bruker Daltonics GmbH, Bremen, Germany) as described previously [16,30]. DNA was extracted from heat-inactivated pure cultures (biomass) using the HighPure PCR Template Preparation Kit (Roche Diagnostics, Mannheim, Germany) according to the manufacturer’s instructions. DNA quantity and purity were determined using a NanoDrop™ 1000 spectrophotometer (Thermo Fisher Scientific, Wilmington, NC, USA). The AMOS-PCR was performed to differentiate *Brucella* species [31,32] followed by a multiplex Bruce-ladder PCR assay for strain and biovar/strain typing [33,34].

### 2.2. Whole-Genome Sequencing

Total genomic DNA was quantified with a Qubit fluorometer (Qubit^TM^ DNA HS assay; Life Technologies Holdings Pte Ltd., Singapore), and library preparation was performed using the Nextera XT library preparation kit (Illumina Inc., San Diego, CA, USA). Sequencing was performed at an Illumina MiSeq targeting 300 bp long reads with 200 bp insertion size.

Quality assessment of the paired-end Illumina sequence data was performed using the qc_pipeline, a bioinformatics workflow that was developed for quick assessment of Illumina reads. The source code is available at https://gitlab.com/FLI_Bioinfo/qc_pipeline (accessed on 14 June 2021). Briefly, FastQC (v 0.11.8, Babraham Bioinformatics, Babraham Institute, Cambridge, UK) was used to check the quality metrics of Illumina sequence data. Mash (v 2.3) (Mash is freely released under a BSD license, The University of California, USA) was used to estimate genome size from k-mer content [35]. Theoretical coverage was calculated by dividing the total number of sequenced bases over the estimated genome size.

To put the sequenced Egyptian strains into an international context, we searched (accessed October 2020) the NCBI Sequence Read Archive (SRA) for paired-end, Illumina sequencing data of *Brucella abortus* and *Brucella melitensis* (Table S1).

Bioinformatics analysis for the characterization and analysis of genomic data was performed using WGSBAC (v 2.0.0), available at https://gitlab.com/FLI_Bioinfo/WGSBAC accessed on 1 December 2020. Briefly, Illumina reads were processed and assembled using the software Shovill (v 1.0.4; https://github.com/tseemann/shovill accessed on 14 December 2020). This software includes steps for adapter trimming by using trimmomatic (v 0.36), overlapping paired-end reads using FLASH (v 1.2), de novo assembly using SPAdes (v 1.34), assembly improvement using pilon (v.122), and filtering contigs that are below 3x k-mer coverage and 500 bp contig length. Quality control of assembled contigs was performed using QUAST (v 5.0.2) [36]. Checks for contamination were performed at the read level and contig level using Kraken2 (v 2.0.7_beta) [37].

Phenotypically, antimicrobial resistant strains of *B. abortus* and *B. melitensis* published in our previous study [16] were analyzed for the presence of any antimicrobial resistance genes or SNPs in the genes *gyr*A, *gyr*B, or *rpo*B responsible for antimicrobial resistance against ciprofloxacin, imipenem, or rifampicin [38,39]. Assembled contigs were searched for antimicrobial resistance genes using Abricate (v 0.8.10) and the databases NCBI AMRFinderPlus (Accession: PRJNA313047), and virulence determinants using the virulence factor database [40], respectively.

MLST profiles were identified for the *Brucella* isolates using MLST (v 2.16.1, https://github.com/tseemann/mlst accessed on 1 June 2021) and MLST scheme “brucella” from pubmlst. in silico MLVA-16 based on assembled contigs was performed as previously described [41] by adapting the tool MISTReSS (https://github.com/Papos92/MISTReSS accessed on 14 December 2020).

SeqSphere + (v 07, Ridom GmbH, Münster, Germany) was used for isolate characterization using the cgMLST typing scheme of *B. melitensis* available on the SeqSphere database (https://www.cgmlst.org/ncs accessed on 1 June 2018), which includes 2704 targets in core genome [1]. Minimum Spanning Tree (MST) based on the allelic differences between *B. melitensis* strains was created using SeqSphere.

Core-genome SNPs (cgSNPs) were identified using snippy (v 4.3.6) and the core genome maximum likelihood tree was built using RAxML (v 8.2.12). As a reference, *B. melitensis* strain 16M (accessions NC_003317 and NC_003318) and *B. abortus* strain 2308 (accessions ASM54005.1) were used. snp-dists (v 0.6.3) was used to calculate the SNP distance between each pair of genomes. Additionally, comparative analysis was performed to check the genetic diversity of *Brucella* isolates of Egyptian and neighboring Mediterranean, African, and Asian countries [27,42] (Table S1). Tree visualization for both in silico MLVA-16 and cgSNP analyses was achieved by using iTOL v4. All generated data were submitted to the National Center for Biotechnology Information (NCBI).

SNP annotation was performed using SnpEff (v 4.3.1t) tool as described [43] to predict the cgSNP coding effects in the genome and to assign the cgSNP variants of the 8 *B. abortus* and 21 *B. melitensis* field isolates to their positions on the two chromosomes (ID NZ_CP007681 with a length of 2,128,683 bp and NZ_CP007680 with a length of 1,160,817 bp) of reference, *B. abortus* and *B. melitensis* 16M (ID NC_003317 and NC_003318), respectively.

To reveal positional genomic variation, Circos software was used for circular presentation of the distribution of missense SNPs, tRNA, and rRNA in chromosomes I and II of *B. abortus* strain BDW, and *B. melitensis* strain 16M [44].

## 3. Results

### 3.1. Identification and Differentiation of Brucella Isolates

Twenty-nine isolates were confirmed as *Brucella* with microbiological/biochemical methods and MALDI-TOF MS (Table S1). Molecular assays (AMOS-PCR and Bruce-ladder PCR) identified 21 *B. melitensis* isolates (17 from cattle, 2 from buffaloes, 1 from a sheep and 1 from a goat) and 8 *B. abortus* isolates (from 7 cattle and a buffalo). PCR assays confirmed these isolates as field strains. Biochemical methods revealed biovar 3 in *B. melitensis* and biovar 1 in *B. abortus*.

### 3.2. Brucella Genome

In this study, 21 genomes of *B. melitensis* and 8 genomes of *B. abortus* were sequenced. The average number of reads was 962,993.9 (min 358,990, max 2,068,092) and 1,194,287.25 (min 449,286, max 1,967,264) for *B. melitensis* and *B. abortus*, respectively (Table S1), which yields an average genome coverage of 69.8 fold (min 26, max 167) and 87.1 fold (range min-max of 31–143), respectively. Genome assembly yielded an average genome size of 3,289,627.4 bp (min 3,278,036 bp, max 3,300,052 bp) and 326,493 bp (min 3,263,728 bp, max 3,265,874 bp) for *B. melitensis* and *B. abortus*, respectively, with N50 average values of 295,160.05 bp and 208,398.9 bp. All generated data were submitted to the National Center for Biotechnology Information (NCBI) and are available at Bioproject: PRJNA650270.

### 3.3. MLST, In Silico MLVA-16, and cgSNP Analysis of B. abortus and B. melitensis Isolates

In silico whole genome classical MLST (Table S1), MLVA-16 [41] and core-genome SNP analysis were applied to study the epidemiological situation in Egypt.

By MLST typing, ST11 was identified in all *B. melitensis* and ST1 in all *B. abortus* strains (Table S1). The ST11 strains were clustered into the Mediterranean lineage, indicating a general phylogenetic relationship of Egyptian *B. melitensis* strains to those from the Mediterranean region. All *B. abortus* were typed into ST1, which was predominantly detected in isolates from bovines and humans from UK, Ireland, New Zealand, Mexico, USA, India, Portugal, Bolivia, and Zimbabwe.

All 8 *B. abortus* bv1 isolates were clustered into one main cluster producing 2 distinct genotypes after in silico MLVA-16 analysis (Table S2a). All loci from panel 1, panel 2A, and panel 2B were monomorphic except for locus *Bruce16* from panel 2B. Two *B. abortus* strains (18RB17256 and 18RB17259) had the same genotype (GT-1) and the remaining six strains (18RB17233, 18RB17242, 18RB17243, 18RB17245, 18RB17255, 18RB17257) shared the same genotype (GT-2).

*Brucella abortus* strain (18RB17256) isolated from cattle in 2017 from Mansoura city, Dakahlia governorate, had the same MLVA-16 genotype (18RB17259) recovered from cattle in 2017 from Toukh town, Qalyubia governorate. *Brucella abortus* strain (18RB17257) isolated from cattle in 2017 from the Sirs El-Layyan area, Monufia governorate, shared the same genotype with the strain 18RB17233 isolated from cattle from the Al-Badrashein area, Giza governorate in 2017, the strains 18RB17242 and 18RB17243 isolated from Beheira (Damanhour) in 2016 from cattle, the strain 18RB17255 recovered from cattle from Beheira (Ad-Dilinjat) governorate in 2017, and the strain 18RB17245 that was obtained from a buffalo in 2017 from Asyut governorate. Additionally, two global *B. abortus* bv1 genotypes (Pond24132 and BAU21/S4023reads) from Spain and Bangladesh respectively, geared from NCBI, formed a separate cluster with their local counterparts. Detailed metadata of *B. abortus* bv1 isolates are given in Tables S1 and S2a.

**Figure 1:**
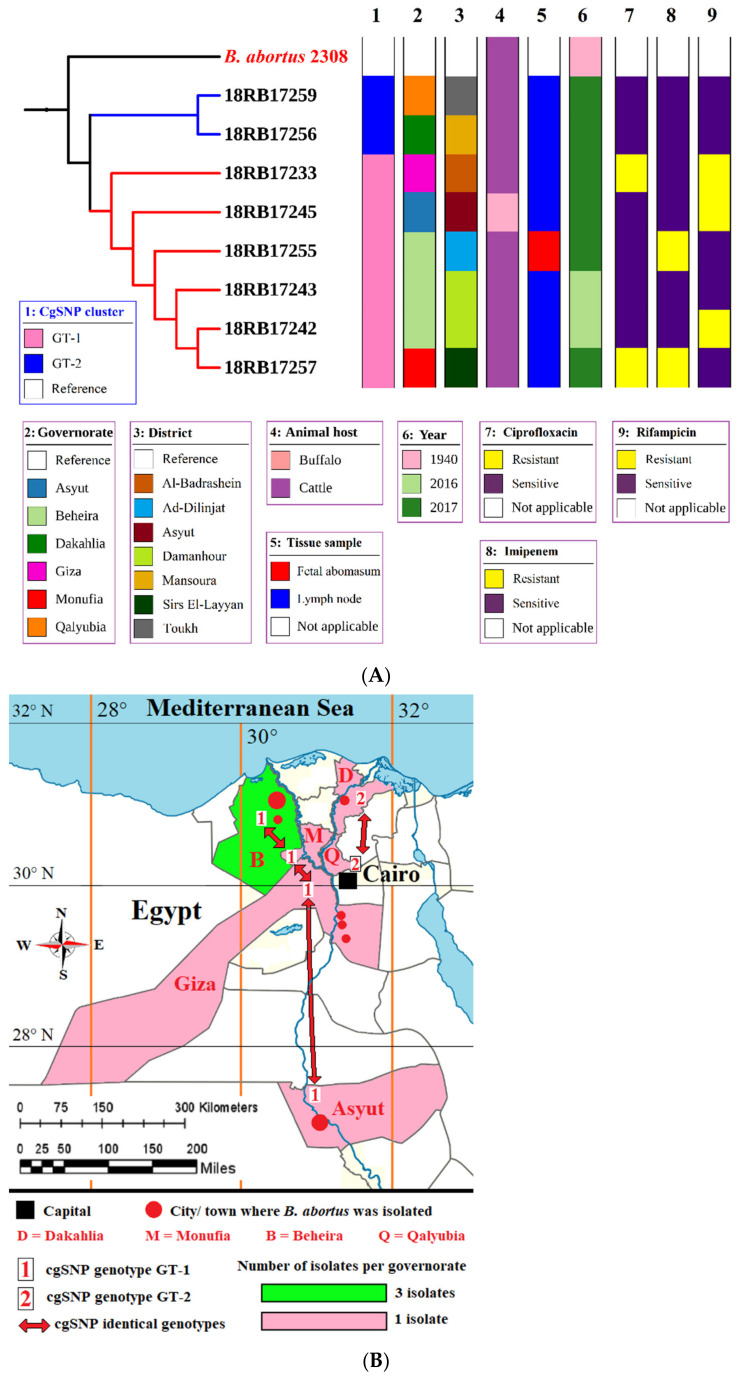
(**A**) Phylogenetic tree based on cgSNPs of the two genotypes of the eight local *B. abortus* bv1 isolates https://itol.embl.de/tree/19322115223541615546544 accessed on 26 May 2021. (**B**) Map of the study area showing the geographic distribution of the two cgSNP genotypes recovered from cattle and a buffalo in six governorates.

**Figure 2:**
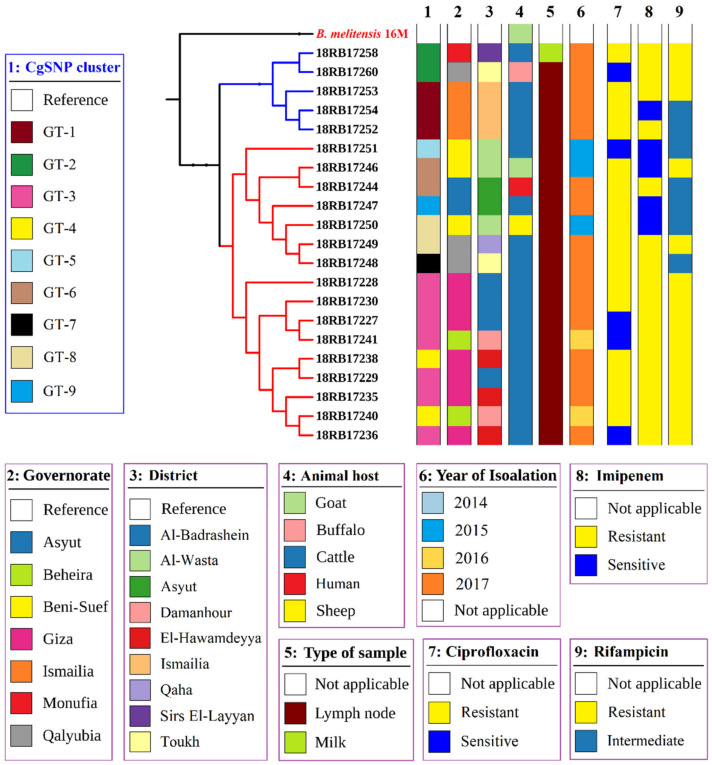
Phylogenetic tree based on cgSNPs of the nine genotypes of the 21 local *B. melitensis* bv3 isolates.

**Figure 3:**
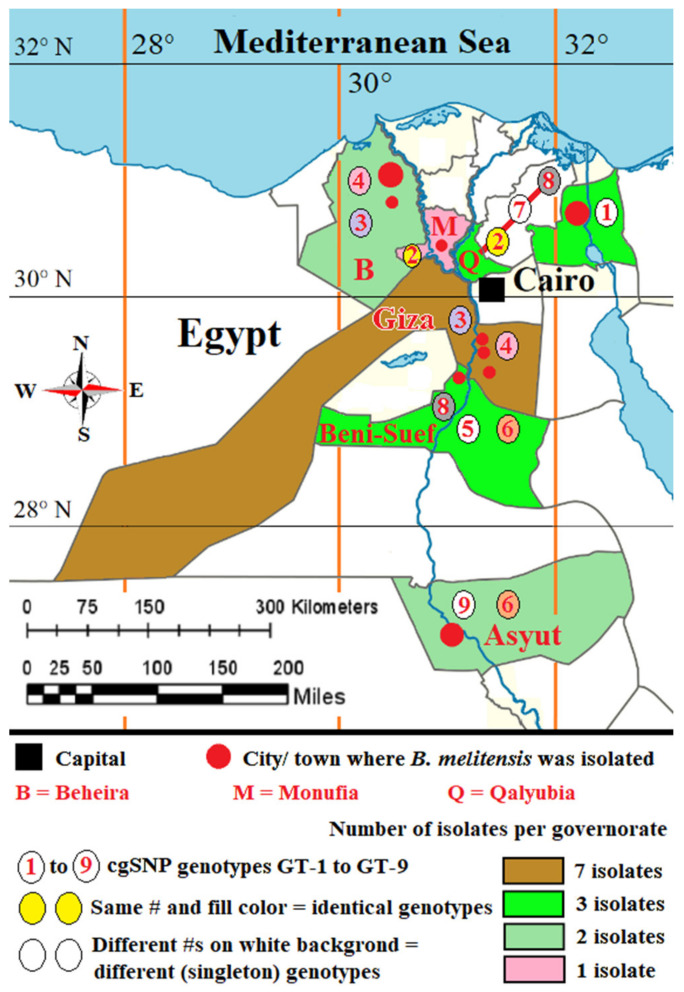
Map of the study area showing the geographic distribution of the aforementioned nine cgSNP genotypes recovered from cattle, buffaloes, sheep, and goats in seven governorates.

The cgSNP analysis for *B. abortus* bases on an alignment of core genes. cgSNP analysis divided the *B. abortus* strains into two genotypes, GT-1 and GT-2 (see Figure 1 and Table S3). *Brucella melitensis* isolates had nine cgSNP genotypes (GT-1 to GT-9), as revealed in Figure 2 and Figure 3.

Six *B. abortus* bv1 isolates (18RB17233, 18RB17242, 18RB17243, 18RB17245, 18RB17255 and 18RB17257) were found to have genotype GT-1 with 0 SNP pairwise differences (Figure 1A,B). They were isolated from cattle and buffaloes from different governorates in Egypt in different years. The isolate 18RB17233 was recovered from cattle from Al-Badrashein area, Giza governorate in 2017. On the other hand, the isolates 18RB17242 and 18RB17243 were recovered from Beheira (Damanhour) in 2016 from cattle. Similarly, the isolate 18RB17255 was obtained from cattle in Beheira governorate but from a different district (Ad-Dilinjat) in 2017. The isolate 18RB17245 was found in a buffalo from Asyut governorate in 2017. The *B. abortus* strain 18RB17257 was isolated from cattle in 2017 from Sirs Al-Layyan area, Monufia governorate. The second genotype (GT-2) contained two identical (0 SNP distance) isolates of *B. abortus* (18RB17256 and 18RB17259) from cattle in 2017 but from different governorates (18RB17256 from Mansoura city, Dakahlia, and 18RB17259 from Toukh town, Qalyubia). It is noteworthy that GT-2 was sensitive to the antibiotics ciprofloxacin, imipenem, and rifampicin, while members of GT-1 revealed variable sensitivities.

The 21 *B. melitensis* bv3 isolates gathered in two clusters (subclusters 1 and 2) with 10 genotypes (GT-1 to GT-10) by in silico MLVA-16 analysis (Table S2b). All loci of the panel-1 and panel-2A except *Bruce18* and *Bruce19* were homogenous. In contrast, the most discriminatory loci were *Bruce18* (Panel 2A) and *Bruce4*, *Bruce7*, *Bruce9*, and *Bruce16* from panel-2B. *B. melitensis* isolates from cattle in 2017 from Al-Badrashein (18RB17227, 18RB17228, 18RB17229, 18RB17230) and Al-Hawamdeyya (18RB17235, 18RB17236, and 18RB17238), Giza governorate shared the same genotype (GT-1) with isolates (18RB17240 and 18RB17241) of *B. melitensis* bv3 recovered from cattle in 2016 from Damanhour city, Beheira governorate. The *B. melitensis* strains isolated from a goat in 2015 (18RB17246, GT-2) from Al-Wasta area, Beni-Suef governorate, was very similar to a strain (18RB17244, GT-3) isolated from a buffalo in 2017 from Asyut governorate with very low genetic diversity (*Bruce09*: 6 to 7). Identical genotypes (GT-4) were found in isolates (18RB17252, 18RB17253, and 18RB17254) from Ismailia governorate isolated from cattle in 2017. *B. melitensis* bv3 strains from Beni-Suef governorate had the same genotype (GT-5) as an isolate from sheep (18RB17250) in 2015 and an isolate of Qalyubia governorate (18RB17249) from cattle in 2017. *Brucella melitensis* strains recovered from cattle (18RB17247, GT-6 isolated in 2017 from Asyut, and 18RB17248, GT-7 isolated in 2017 from Toukh, Qalyubia) had very low genetic similarity (*Bruce04*: 6 to 7 and *Bruce09*:9 to 10). One *B. melitensis* strain isolated from cattle (18RB17251, GT-8) in 2015 from Al-Wasta, Beni-Suef was of a distinct genotype. A GT-9 strain (18RB17258) isolated from cattle in 2017 from Sirs El-Layyan, Monufia was a distinct genotype from *B. melitensis* bv3 strain 18RB17260 (GT-10), isolated from a buffalo in 2017 from Toukh, Qalyubia. Detailed metadata of *B. melitensis* bv3 strains are given in Table S2. The cgSNP analysis for *B. melitensis* is based on an alignment of 2977 core genes. The number of pairwise core-genome SNPs between Egyptian isolates ranges between 0 and 119 (Table S3). The inferred phylogeny splits the isolates into 2 main clusters containing nine genotypes, GT-1 to GT-9 (Figure 2 and Table S3). Cluster 1 involved five isolates (18RB17252, 18RB17253, 18RB17254, 18RB17258, and 18RB17260) out of the 21 *B. melitensis* isolates. The *B. melitensis* isolates (18RB17252, 18RB17253, 18RB17254) were of identical (0 SNP distance) genotypes (GT-1) to those recovered from cattle in 2017 from Ismailia governorate (Figure 2, Figure 3 and Table S3). A *B. melitensis* strain (18RB17258, GT-2) in cluster 1, isolated from cattle in 2017 from the Sirs Al-Layyan district of Monufia governorate, was 40 SNP distant from a strain (18RB17260) recovered from a buffalo in 2017 from Toukh town, Qalyubia. Cluster 2 contained 16 *B. melitensis* isolates (18RB17227, 18RB17228, 18RB17229, 18RB17230, 18RB17235, 18RB17236, 18RB17238, 18RB17240, 18RB17241, 18RB17244, 18RB17246, 18RB17247, 18RB17248, 18RB17249, 18RB17250 and 18RB17251). Seven isolates from cluster 2 (18Rb17227, 18RB17228, 18RB17229, 18RB17230, 18RB1717235, 18RB17236, and 18RB17241) were similar (GT-3) to those isolated from cattle in 2017 from Giza governorate with the exception of one isolate (18RB17241) from Beheira governorate that was detected in 2016. Two *Brucella melitensis* strains (18RB17238, and 18RB17240) were almost identical (4 SNP distance) genotypes (GT-4), strain 18RB17238 was isolated from cattle in 2017 from Al-Badrashein and Al-Hawamdeyya districts of Giza governorate, respectively, while strain 18RB17240 was recovered from cattle in 2016 from Damanhour city, Beheira governorate. Another strain (18RB17251, GT-5) from the same cluster (cluster 2) was a singleton genotype isolated from cattle in 2015 from the Al-Wasta area, Beni-Suef governorate.

**Figure 4:**
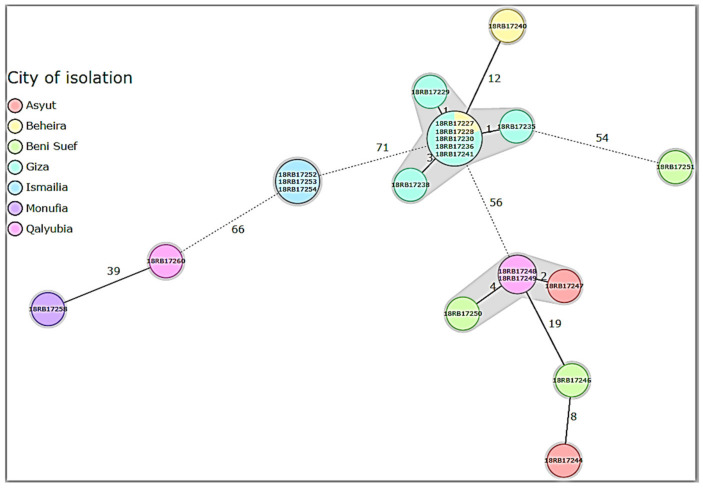
Minimum Spanning Tree (MST) based on the allelic differences between 21 *B. melitensis* strains. The cgMLST scheme developed for *B. melitensis* was used. Unique cgMLST allelic profiles are represented by nodes (circles). Numbers on connecting lines between the nodes refer to the number of differing alleles.

Two highly related *B. melitensis* isolates, 18RB17244 and 18RB17246 of GT-6, with a difference of only 5 SNPs were recovered from a buffalo and a goat in 2017 and 2015 from Asyut and Beni-Suef governorates, respectively (Figure 3). Minimum Spanning Tree (MST) based on the allelic differences between 21 *B. melitensis* strains using SeqSphere v07 showed the genetic diversity of the isolated 21 *B. melitensis* (Figure 4). Furthermore, two *B. melitensis* genotypes (18RB17248, GT-7, and 18RB17249, GT-8) showing low genetic diversity with a difference of only 6 SNPs were isolated from cattle in 2017 from Toukh and Qaha districts of Qalyubia governorate, respectively. The *B. melitensis* genotype 9 (18RB17247, GT-9) isolated from cattle varied by 24 SNPs from another genotype (18RB17244, GT-6) recovered from buffalo in 2017 in the same governorate (Asyut). The *B. melitensis* genotype 8 (18RB17250, GT-8) isolated from a sheep varied by 24 SNPs from another genotype (18RB17246, GT-6) recovered from a goat in 2015 from Al-Wasta district of Beni-Suef governorate.

Genotype GT-1 of Ismailia governorate showed maximum variation up to 99 SNPs from genotypes GT-3 and GT-4 of Giza. GT-1 of Ismailia was also 95 SNPs different from the singleton genotype GT-7 of Qalyubia. Genotypes GT-3 and GT-4 of Giza were different in up to 98 SNPs as compared to genotypes GT-2 and GT-8 of Qalyubia and in 65 to 67 SNPs upon comparison with the singleton genotype GT-5 of Beni-Suef. The singleton genotype GT-5 differed in 82 SNPs with the singleton genotype GT-7 of Qalyubia. The singleton genotype GT-5 of Beni-Suef varied in up to 107 SNPs from genotypes GT-2 and GT-8 of Qalyubia and in 79 SNPs from the singleton genotype GT-9 of Asyut. The genotypes GT-3 and GT-4 of Beheira, identical to Giza genotypes GT-3 and GT-4, are up to 4 SNPs different from each other. Other genotypes showed less SNP difference.

### 3.4. Comparison of Local Brucella Genotypes with Selected Genotypes from Neighboring Countries Using In Silico MLVA-16 and cgSNP Analyses

**Figure 5:**
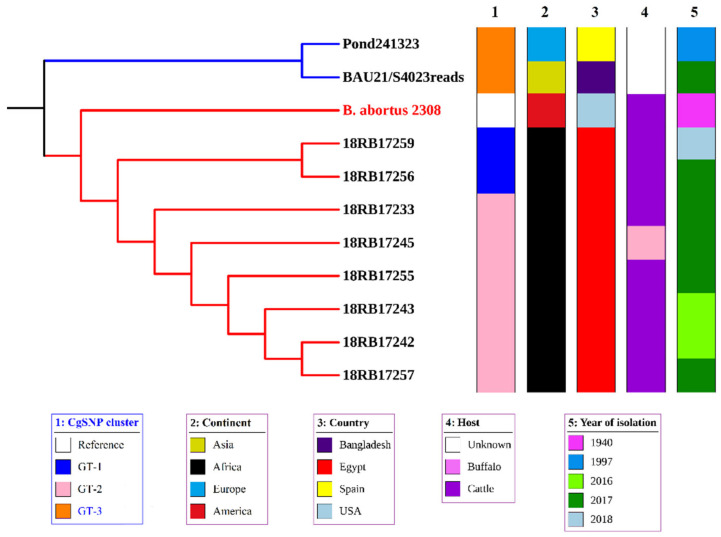
Phylogenetic tree based on cgSNPs of the two local *B. abortus* bv1 genotypes as linked to selected global genomes of *B. abortus* bv1 from the Mediterranean littoral, Africa, and Asia available at the NCBI Genome Assembly and Annotation.

The isolated strains of *B. abortus* in our study revealed ST 1, while the strain from a Mediterranean country like Spain revealed ST-2. The in silico MLVA-16 profile classified the Spanish and Bangladeshi genotypes into a separate cluster and the two Egyptian *B. abortus* genotypes of the 8 isolates in another cluster (Table S2a). cgSNP analysis revealed difference of genotypes by more than 700 SNPs from *B. abortus* strains from the two global *B. abortus* bv1 genotypes geared from NCBI (Pond24132 and BAU21/S4023reads) from Spain and Bangladesh respectively that were included in one cluster with local genotypes GT-1 (18RB17233) and GT-2 (18RB17256 and 18RB17259) and the reference *B. abortus* 2308 (Figure 5). The other local *B. abortus* isolates belonging to genotypes GT-1 formed a separate cluster.

**Figure 6:**
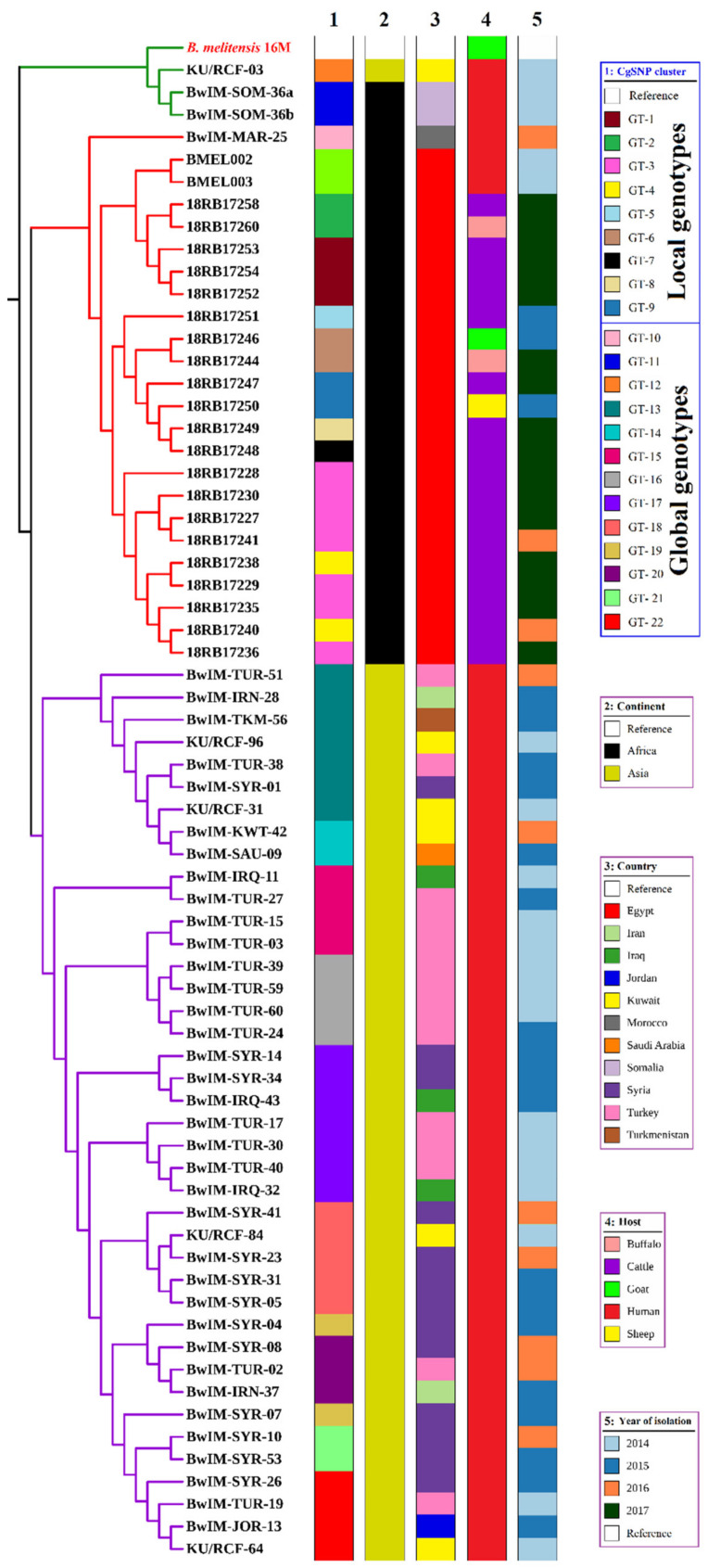
Phylogenetic tree based on cgSNPs of the local 9 *B. melitensis* bv3 genotypes as linked to selected global genomes of *B. melitensis* bv3 from the Mediterranean littoral, Africa, and Asia available at the NCBI Genome Assembly and Annotation.

The 21 Egyptian *B. melitensis* bv3 isolates were compared with corresponding global genotypes from neighboring countries. A total of 44 genotypes from Iran, Iraq, Jordan, Kuwait, Morocco, Saudi Arabia, Somalia, Syria, Turkey, Turkmenistan, and Egypt were downloaded and analyzed. All Egyptian strains revealed ST 11, including the two downloaded human strains. In correspondence, these two human strains cluster together with Egyptian animal strains in cgSNP analysis (Figure 6). All strains of the Egyptian cluster are at least 500 cgSNPs away from strains outside Egypt (Table S3). One Moroccan genotype (BwIM-MAR25) formed a different clade. A Kuwaiti genotype (KU/RCF-03), together with the 2 Somali genotypes (BwIM-SOM-36a and BwIM-SOM-36b), formed an independent cluster, while the other genotypes from Asia formed a big cluster with eight sub-clusters.

### 3.5. SNP Annotation

Analysis of the cgSNPs of the eight *B. abortus* isolates revealed that 256 SNPs were divided between chromosome 1, with 142 SNPs, and chromosome 2, with 114 SNPs. The corresponding analysis of the 21 *B. melitensis* isolates indicated that 2983 SNPs were divided between chromosome 1, with 1872 SNPs, and chromosome 2, with 1111 SNPs. The SNPs were distributed among different annotation types as shown in Figure S1a–d and Table S4.

### 3.6. Factors for Antimicrobial Resistance and Virulence

Resistance Gene Identifier (RGI), available at the Comprehensive Antibiotic Resistance Database (CARD), identified two genes (*Brucella suis-*mprF and TriC) with 100% identity within its available database involved in antimicrobial resistance mechanisms. The tool also identified *Bifidobacterium adolescentis rpo*B conferring resistance to rifampicin. However, AMRFinderPlus (NCBI) and RGI (CARD) did not identify any of the proposed genes responsible for antimicrobial resistance against macrolides (*erm, mef, msr*), tetracyclines (*tet* genes), beta-lactams (*mec*A), or trimethoprim (*fol*A).

A point mutation was identified in three isolates of *B. melitensis* (18RB17252, 18RB17253, and 18RB17254) in the *rpo*B gene at position 2784-CGC to CGT/Arg to Arg. Another mutation was detected in the *rpo*B of *B. melitensis* strain (18RB17260) at position 2394-ACG to ACT/Thr to Thr (Table S5). One point mutation was identified in the *gyr*A gene of *B. melitensis* strains (18RB17252, 18RB17253, and 18RB17254) at 297-GAT to GAA/Asp to Glu (Table S5).

In total, 43 virulence genes were identified in each of the *B. melitensis* and *B. abortus* isolates (Table S5). These genes are mainly responsible for host immune evasion, intracellular survival, regulation, and expression of the Type IV secretion system in brucellae. Genes responsible for lipopolysaccharide (LPS) synthesis, maturation, and functioning were identified in *B. melitensis* and *B. abortus* isolates.

## 4. Discussion

Brucellosis is a zoonotic disease of public health importance characterized by reproductive losses in animals. It is a debilitating disease in humans and is still endemic in many countries including Egypt. In this study, molecular characterization and genotyping of *Brucella* isolates from cattle, buffaloes, sheep, and goats obtained from different geographical locations in Egypt were performed. Additionally, the assessment of antimicrobial resistance genes and virulence factors based on whole-genome sequencing of *Brucella* isolates is reported. These results contribute to a better understanding of the transmission and geographic spread of brucellae among livestock in Egypt and pave the way for specific treatment and control of the disease in animals and humans as well. The estimated annual incidence of brucellosis in Egypt per 100,000 population was 64 and 70 in 2002 and 2003, respectively [45].

Both *B. abortus* and *B. melitensis* are circulating within the animal population as they have been recovered from cattle and buffaloes, sheep and goats, and dogs and cats [12,17,46,47]. The isolation of *B. melitensis* from cattle and buffaloes confirms its ability to establish permanent animal reservoirs in atypical hosts [8,17]. Circulation in cattle and buffaloes of *B. melitensis*, the most virulent of all brucellae, increases the risk for occupational human infection as most animals are owned by individual households and are kept in small, usually mixed herds that move daily between house yards and grazing grounds. *Brucella melitensis* was identified in lymph nodes and milk samples (Table S1) from preference (one from a sheep and one from a goat) and occasional hosts (17 cattle and 2 buffaloes). The bovine infection with *B. melitensis* represents a serious public health problem as most of the milk is produced by cattle and buffaloes, and this can be a potential source of human infection. Additionally, these animals may lead to environmental contamination as a result of abortions and the birth of infected calves. The isolation of *B. abortus* from cats and dogs also shows the role of carrier hosts in the dissemination and re-emergence of *Brucella* spp. [46]. Identification of brucellae, particularly *B. suis* and *B. melitensis*, from pigs highlighted the endemicity of the agent in the country [16]. This confusing epidemiological situation may result in difficulties for effective surveillance and control of brucellosis.

*Brucella melitensis* is the predominant species in Egypt, as it is identified in almost every animal species as well as in humans [8,9,17]. The high prevalence of *B. melitensis* bv3 in large ruminants (secondary host) in the country represents a complex epidemiological situation that necessitates detailed epidemiological investigations and genotyping of prevalent strains. *Brucella abortus* was identified from the lymph node and fetal stomach contents. These isolates were recovered from preference hosts (seven from cattle and one from a buffalo).

Brucellosis control largely relies on efficient diagnosis and epidemiological assessment of prevailing *Brucella* species in a particular region. The genus *Brucella* is a highly homogenous and highly monomorphic group of bacteria with minimal genetic variations. Routine culture isolation and serological and current molecular assays help in disease diagnosis but are unable to differentiate and trace-back circulating genotypes [8,17,22]. MALDI-TOF MS reliably identified *Brucella* isolates at the genus level, with less reliable sub-genus recognition. The advantages of this method, compared to conventional techniques, are rapidity, cost-effectiveness, accuracy, and suitability for the high-throughput identification of bacteria [48]. The disadvantages of MALDI-TOF-MS can be overcome when potential taxonomic and genetic inconsistencies are considered during the generation of the reference library [49]. This is mainly because of the low abundance and poor ionization efficiency of analytes (peptides) and the coexistence of plentiful interfering chemical species in the sample matrices. Scientists are currently solving this problem by selective enrichment techniques of the sample [50].

Several studies have proven the usefulness of MLVA in the identification of *Brucella* genotypes along with their epidemiological monitoring and tracing back of the source of infection [8,17,25]. However, this method showed some limitations, notably hyper-variability of some VNTR loci and homoplasy (convergent evolution). Thus, it is currently suggested that MLVA be replaced with WGS-based molecular tools to provide better resolution in discriminating genotypes [1,51].

According to in silico MLVA-16 profiles, all tested *B. melitensis* and *B. abortus* showed complete homogeneity in the panel 1 markers used for species assignment (Table S2a,b). No difference was observed in isolates collected from different animals in different governorates, thus exhibiting the typical Egyptian clusters [17]. Loci from *Bruce16*-Panel 2B in *B. abortus* profiles were found to be discriminatory. Similarly, the loci (*Bruc04*, *Bruc09*, and *Bruce16*) of panel 2B distinguished *B. melitensis* isolates into different genotypes.

Although Illumina based MLVA-16 typing is easy and cheap, it revealed some limitations in the in silico MLVA-16 analysis. Two loci, *Bruce21* (Panel 2A) and *Bruce07* (Panel 2B) were missing in 2 and 7 isolates of *B. abortus* and *B. melitensis*, respectively. These missing values may be attributed to the incomplete genomes of *B. melitensis* produced by Illumina sequencing. However, for the closed (reference) genomes in silico, MLVA-16 analysis worked properly [17,27,41]. in silico MLVA-16 genotypic analysis of *B. abortus* and *B. melitensis* collected from cattle, buffaloes, sheep, and goats revealed 2 and 10 genotypes, respectively.

The *B. abortus* genotype 1 (GT-1) circulating in cattle from Dakahlia and Qalyubia governorates highlighted the animal movement and spread of the brucellae (Table S2a). Similarly, other genotypes (GT-2) of *B. abortus* were identified in cattle and buffaloes from Asyut, Beheira, Giza, and Monufia governorates. MLVA-16 analysis identified 10 genotypes of *B. melitensis* recovered from cattle, buffaloes, sheep and goats (Table S2b). GT-1 of *B. melitensis* were exclusive to Giza and Beheira governorates. Genotypes 2, 3, 5, 6, 7, 8, 9, and 10 recovered from cattle and buffaloes from Beni-Suef, Asyut, Qalyubia, Asyut, Qalyubia, Beni-Suef, Monufia, and Qalyubia governorates respectively were singletons. Qalyubia and Monufia governorates had more heterogenous *B. melitensis* genotypes circulating in animals. Genotypes 2 and 5 were recovered from goats and sheep respectively from different governorates.

MLVA profile highlighted the genetic diversity of *B. melitensis* strains in Egypt. Genotyping analysis using MLVA-15 and MLVA-16 were performed in *B. abortus* and *B. melitensis* strains recovered from humans and animals in Egypt previously [8,12,17,52]. In this study, six isolates of *B. abortus* from 4 governorates (Beheira, Monufia, Giza and Asyut) belonging to GT-2 shared an MLVA-16 profile with a formerly reported strain (Egypt-66) from a buffalo in Ismailia governorate [17], indicating the spread of this genotype. Two genotypes (GT-7 and GT-8) of *B. melitensis* strains isolated from cattle at Qalyubia and Beni-Suef governorates in the present study were very closely related to previously reported strains 23-Bm3-Suef and 36-Bm3-Suef from cattle in Beni-Suef governorate [8]. Thus, in silico MLVA-16 was a molecular epidemiological tool that proved to be useful for the Egyptian setting but needs minimization of chemicals to be more robust.

Core-genome single-nucleotide polymorphism (cgSNP) analysis revealed 2 and 9 genotypes of *B. abortus* and *B. melitensis*, respectively recovered from cattle, buffaloes, sheep and goats from various governorates in different years (Figure 1A and Figure 2). Each of the MLVA-16 and cgSNP profiles detected 2 genotypes of *B. abortus*. However, cgSNP discriminated *B. melitensis* into 9 genotypes versus 10 genotypes (Figure 2, Tables S2b and S3). Two MLVA genotypes (18RB17244-Asyut Egy and 18RB17246-Beni-Suef Egy) were identified as a single cgSNP genotype. Likewise, another 2 MLVA genotypes (18RB17258-Monufia Egy and 18RB17260-Qalyubia Egy) had one cgSNP pattern. The MLVA genotype present in both Beheira and Giza was further split into 2 different cgSNP genotypes. The 3 MLVA genotypes of *B. melitensis* in Beni-Suef were still identified by cgSNP as 3 genotypes, but the demarcation was evident in classifying one of the genotypes as distinct from a genotype in Qalyubia that was identical by MLVA. Genotypes from other governorates remained the same in terms of their number. The resolution power of cgSNP versus in silico MLVA-16 and MLST is attributed to the fact that there are thousands of SNPs scattered throughout the *Brucella* genome as compared to about a few hundred tandem repeat loci [1,27,41].

SNP profiling also provides deeper insight into the genome structure of an isolate as SNP annotation categorizes SNP sequence variants based on their relationship to coding sequences in the genome and how they may change the coding sequence and affect the gene product. Thus, SNP variants can be linked to phenotypic changes. Investigating the SNP types of the two chromosomes of *B. abortus* (Figure S1a,b) have the same three major SNP types as *B. melitensis* (Figure S1c,d), which are missense, synonymous, and upstream gene variants. Missense variants had the highest percentage in the two chromosomes of *B. abortus* as they increased from chromosome 1 to chromosome 2 (43.7% to 52.6%), Figure S2a,b, while their number were nearly the same (62 and 60). Referring to Figure S2c,d of *B. melitensis*, it is generally noted that the four categories of missense variants, synonymous variants, upstream gene variants, and stop gained almost maintained the same percentages across the two chromosomes within a deviation of ±2%, while the other categories were unique to each chromosome. Missense variants, leading to changes in the corresponding amino acids, had the highest percentage among all SNP types and their percentages showed a slight increase from chromosome 1 to chromosome 2 (47.06% to 48.06%), despite the significant drop of their number from 881 to 534. SNP missense variants recorded an increase in the percentages from chromosome 1 to chromosome 2 in both *B. abortus* and *B. melitensis*. This increase was significant in *B. abortus* but very small in *B. melitensis*. The two *Brucella* species had a decrease in numbers between the two chromosomes whether in total or individual types.

The findings suggest that different genotypes of brucellae are heterogeneously circulating in the country. Uncontrolled movement of animals among various governorates, as animals are cheaper in certain governorates than others, definitely facilitates the spreading of brucellae from one region to another. Open and mixed animal markets countrywide result in the spread of various diseases including brucellosis [17]. It is noteworthy that the same cgSNP genotype GT-1 of *B. abortus* bv1 was found in Beheira and Asyut, almost 560 km apart. This finding reflects the chaotic movement of livestock among governorates. To the best of our knowledge, this is the first study highlighting the epidemiology of brucellosis in Egypt using WGS-based cgSNP analysis, which proved to be the most efficient typing tool used.

Comparative genomic analysis of brucellae isolated from Egypt with other global isolates revealed huge differences for *B. abortus* genotypes (Figure 5). *B. abortus* genotypes are unique and prevailing in Egypt, but the limited availability of *B. abortus* genome sequences from neighboring countries hampers complete assessment. Likewise, local *B. melitensis* genotypes were remarkably different from their global counterparts and were very closely related to Egyptian genotypes (Figure 6) from a previous study [27].

In this study, the resistance genes and mutations were analyzed using WGS data to evaluate phenotypic results and to investigate possible genetic markers that could predict resistance. One SNP variant was detected in *gyr*A, a gene associated with ciprofloxacin resistance, while two SNP variants were identified in *rpo*B, a gene associated with rifampicin resistance [53] in 3 and 4 isolates of *B. abortus* and *B. melitensis,* respectively. The point mutation at position 297 (GAT to GAA) in the *gyr*A gene leads to an amino acid change from aspartic acid (Asp) to glutamic acid (Glu), Table S5. The point mutations in *rpo*B gene revealed silent mutations at positions 2784 (CGC to CGT/Arg-Arg) and 2394 (ACG to ACT/Thr-Thr). These alterations are different from mutations previously described as a cause of rifampicin resistance in *Brucella* [38]. Similarly, the mutations in *gyr*A gene were also different from the mutations described previously [54]. However, mutations in the *rpo*B and *gyr*A genes of *B. melitensis*, described formerly, were observed in mutant strains. The SNP changes, therefore, do not seem to be associated with phenotypic rifampicin resistance. However, these findings question whether the recommended broth microdilution method by the CLSI or EUCAST [55,56], and to some extent the gradient strip method, might overestimate in vitro rifampicin resistance in *B. melitensis*. This topic needs to be further addressed in larger multicentre studies. However, the identification of *Bifidobacterium adolescentis rpo*B conferring resistance to rifampicin may be involved in higher phenotypic resistance in these brucellae [57]. None of the proposed antimicrobial resistance genes responsible for resistance against macrolides (*erm, mef, msr*), tetracyclines (*tet* genes), beta-lactams (*mec*A), or trimethoprim (*flo*A) in *Brucella* was detected [53]. The enhanced phenotypic resistance in the *Brucella* isolates of this study may be a result of the efflux of antimicrobials or other unknown mechanisms.

*Brucella suis-mpr*F and *Tri*C genes were identified in all *Brucella* isolates. Many bacterial pathogens achieved resistance to defensin-like cationic antimicrobial peptides (CAMPs) by the multiple peptide resistance factor (MprF) protein. MprF plays a crucial role in *Staphylococcus aureus* virulence and it is involved in resistance to the CAMP-like antibiotic daptomycin [58]. MprF is responsible for the modification of anionic phosphatidylglycerol with positively charged L-lysine, which results in the repulsion of CAMPs. This protein is also known to affect susceptibility to antimicrobials (methicillin, oxacillin, bacitracin, gentamicin, beta-lactams) and other cationic peptides. It is also resistant to moenomycin, susceptible to vancomycin, resistant to human defensins (HNP1-3), and evades oxygen-independent neutrophil killing [59]. The identification of this protein may suggest its involvement, especially in intracellular survival and repulsion of cationic antimicrobials, in *Brucella*. This protein is present in the *B. suis* genome, but not much is known about its role in virulence and antimicrobial resistance in brucellae [60]. TriC is a resistance nodulation cell division (RND) transporter that is part of TriABC-OpmH, a triclosan-specific efflux protein. It has been identified in *Pseudomonas aeruginosa*, which contains two membrane fusion proteins, TriA and TriB [61]. Its function in *Brucella* is not known; however, increased phenotypic resistance against ciprofloxacin and imipenem may suggest that the role of the efflux pump, as described earlier, in *Brucella melitensis* is to confer resistance against quinolones [62].

Brucellae are intracellular, facultative bacteria that can avoid killing mechanisms and proliferate within the cells of the reticuloendothelial system of the host. The pathogenesis of brucellae involves adhesion, invasion, establishment, and dissemination within the host. Several studies have focused on the virulence factors in brucellae directed at the involvement of the outer membrane. The outer membrane contains lipopolysaccharide (LPS), which is the major virulence factor of *Brucella* [63]. It possesses a peculiar non-classical LPS as compared to the classical LPS from *Enterobacteriaceae*, such as *Escherichia coli* [64].

In this study, we identified a set of genes (*lps*B/*lpc*C, *lpx*C, *lpx*D, *fab*Z, *lpx*A, *lpx*B, *kds*A, *kds*B, *pgm*, *gmd*, *per*, *wzm*, *wzt*, *wbk*B, *wbk*C, *wbp*L, *acp*XL, *lpx*E, *lps*A, *htr*B, *acp*XL, *wbo*A, *wbd*A, *wbp*Z, *man*AoAg, *man*CoAg, *pmm*, *wbk*A) regulating the LPS synthesis and its functions (Table S5). These genes were reported previously in brucellae [65]. LPS facilitates brucellae pathogenesis by countering the innate immune defense correlating with poor myeloid differentiation-2 (MD2) binding and low endotoxicity. Additionally, it limits the complement deposition, activation and killing by several neutrophils [66]. The TIR domain-containing proteins BtpA/Btp1/TcpB and BtpB interfere with toll-like receptor (TLR) signaling to temper the host inflammatory response [67]. In addition to LPS, the type IV secretory system (T4SS) plays an important role in adherence of the bacterium to the host cell and cell entry as well as intracellular trafficking and survival [64,68]. Cyclic ß-1, 2-glucans (CßGs) also interfere with cellular trafficking and prevent the phagosome-lysosome fusion cycle. The brucellae containing vesicle (BCV) does not fuse with lysosomes, instead, it interacts with the endoplasmic reticulum (ER), leading to the creation of a specialized vacuole in which the bacteria multiply [69]. The fusion between the endoplasmic reticulum and the BVC depends on the *Brucella vir*B encoded T4SS. Genes responsible for TIR domain-containing proteins (*btp*A and *btp*B), T4SS (*vir*B1-*vir*B12), and CßGs (*cgs*) were identified in this study as reported previously [65]. RicA (Rab2 interacting conserved protein A) was also identified in this study. The RicA-Rab2 interaction may affect the maturation of the BCV in a way that slows intracellular replication, thereby contributing to the evasion of the innate immune system [70].

## 5. Conclusions

This study found WGS based MLVA-16 and core-genome SNP typing to be suitable tools for trace-back analysis of *B. abortus* and *B*. *melitensis* for brucellosis in Egypt. in silico MLVA-16 analysis found 2 *B. abortus* and 10 *B. melitensis* genotypes, while cgSNPs analysis identified 2 *B. abortus* and 9 *B. melitensis* genotypes in a strain collection of 29 isolates. Hence, short read sequencing results in gaps in hard-to-sequence regions and hard-to-reconstruct genomes with bioinformatic software hamper typing. Strains with similar genotypes isolated from different governorates reflect the long endemicity of brucellosis in Egypt with earlier dispersion of types and great local genetic diversity.

Genomic comparison of local and global genotypes of *Brucella* exhibited the uniqueness of Egyptian genotypes of both *B. abortus* and *B. melitensis*. The fast development of antimicrobial resistance highlights the importance of controlling the use of antibiotics for the treatment of brucellosis and other diseases in the country.

To the best of the authors’ knowledge, this is the first WGS based investigation of *Brucella* isolates from livestock belonging to different zones in Egypt.

## Data Availability

The data presented in this study are available within the article or supplementary material.

## Supplementary Materials

The following are available online at https://www.mdpi.com/2076-0817/10/6/759/s1: Table S1. Metadata microbiological and molecular identification and genome characteristics of Brucella spp. isolated from animal species in Egypt versus neighboring countries geared from NCBI. MLST typing of B. abortus and B. melitensis using 7 housekeeping genes; Table S2a,b. In silico MLVA-16 pattern of the local 8 B. abortus bv1 and the 21 B. melitensis bv3 genotypes in comparison with their available global counterparts; Table S3. Core genome SNP distance in B. abortus and B. melitensis isolates from livestock in Egypt; Table S4. SNP variant types for reference (a) B. abortus BDW chromosome 1 (NZ_CP007681) and chromosome 2 (NZ_CP007680), and (b) B. melitensis 16M chromosome 1 (NC_003317) and chromosome 2 (NC_003318); Table S5. Whole-genome sequencing detection of virulence factors in B. abortus and B. melitensis isolates from livestock in Egypt; Figure S1a–d. Circular presentation of the distribution of missense SNPs, tRNA, and rRNA (a) in chromosome I and (b) chromosome II of B. abortus strain BDW, and (c) in chromosome I and (d) chromosome II of B. melitensis strain 16M; Figure S2a–d. Percentages of SNP types for (a) chromosome I and (b) chromosome II of reference B. abortus BDW, and (c) chromosome I and (d) chromosome II of reference B. melitensis 16M.
