# Supplementary material for: Whole-Genome Sequencing for Tracing the Genetic Diversity of Brucella abortus and Brucella melitensis Isolated from Livestock in Egypt
Source: Pathogens. 2021 Jun 16;10(6):759. doi: 10.3390/pathogens10060759 (PMC8235727; doi:10.3390/pathogens10060759)
Supplement: Supplementary file 1 [file pathogens-10-00759-s001.zip › Table S2. B.pdf]

Table S2a. *B. abortus* isolates recovered from livestock examined by MLVA-16 analysis. Panel 1 includes 8 minisatellite markers and panel 2 (2A and 2B) another eight microsatellites.

ID	Strains	MLVA type																
		Panel 1								Panel 2A			Panel 2B					MLVA type
		Bruce06	Bruce08	Bruce11	Bruce12	Bruce42	Bruce43	Bruce45	Bruce55	Bruce18	Bruce19	Bruce21	Bruce04	Bruce07	Bruce09	Bruce16	Bruce30	
18RB17233	*B. abortus*	4	5	4	12	2	2	3	3	6	43	8	4	4	3	4	4	
18RB17242	*B. abortus*	4	5	4	12	2	2	3	3	6	43	8	4	4	3	4	4	
18RB17243	*B. abortus*	4	5	4	12	2	2	3	3	6	43	8	4	4	3	4	4	
18RB17245	*B. abortus*	4	5	4	12	2	2	3	3	6	43	8	4	4	3	4	4	
18RB17255	*B. abortus*	4	5	4	12	2	2	3	3	6	43	8	4	4	3	4	4	
18RB17256	*B. abortus*	4	5	4	12	2	2	3	3	6	43	8	4	4	3	5	4	
18RB17257	*B. abortus*	4	5	4	12	2	2	3	3	6	43	8	4	4	3	4	4	
18RB17259	*B. abortus*	4	5	4	12	2	2	3	3	6	43	8	4	4	3	5	4	
**Reference**	*B. abortus*-2308	4	5	4	12	2	3	3	3	6	43	8	3	7	3	3	5	

Table S2b. *B. melitensis* isolates recovered from livestock examined by MLVA-16 analysis. Panel 1 includes 8 minisatellite markers and panel 2 (2A and 2B) another eight microsatellites.

**ID**	**Strains**	**MLVA type**																
		Panel 1								Panel 2A			Panel 2B					**MLVA type**
		Bruce06	Bruce08	Bruce11	Bruce12	Bruce42	Bruce43	Bruce45	Bruce55	Bruce18	Bruce19	Bruce21	Bruce04	Bruce07	Bruce09	Bruce16	Bruce30	
18RB17227	*B. melitensis*	3	5	3	13	1	1	3	3	7	43	8	5	7	9	11	3	
18RB17228	*B. melitensis*	3	5	3	13	1	1	3	3	7	43	8	5	NA	9	11	3	
18RB17229	*B. melitensis*	3	5	3	13	1	1	3	3	7	43	8	5	NA	9	11	3	
18RB17230	*B. melitensis*	3	5	3	13	1	1	3	3	7	43	8	5	7	9	11	3	
18RB17235	*B. melitensis*	3	5	3	13	1	1	3	3	7	43	8	5	NA	9	11	3	
18RB17236	*B. melitensis*	3	5	3	13	1	1	3	3	7	43	8	5	NA	9	11	3	
18RB17238	*B. melitensis*	3	5	3	13	1	1	3	3	7	43	8	5	NA	9	11	3	
18RB17240	*B. melitensis*	3	5	3	13	1	1	3	3	7	43	8	5	NA	9	11	3	
18RB17241	*B. melitensis*	3	5	3	13	1	1	3	3	7	43	8	5	7	9	11	3	
18RB17244	*B. melitensis*	3	5	3	13	1	1	3	3	8	43	8	7	5	7	4	3	
18RB17246	*B. melitensis*	3	5	3	13	1	1	3	3	8	43	8	7	5	6	4	3	
18RB17247	*B. melitensis*	3	5	3	13	1	1	3	3	8	43	8	7	5	9	5	3	
18RB17248	*B. melitensis*	3	5	3	13	1	1	3	3	8	43	8	6	5	10	5	3	
18RB17249	*B. melitensis*	3	5	3	13	1	1	3	3	8	43	8	6	5	9	5	3	
18RB17250	*B. melitensis*	3	5	3	13	1	1	3	3	8	43	8	6	5	9	5	3	
**18RB17251**	***B. melitensis***	**3**	**5**	**3**	**13**	**1**	**1**	**3**	**3**	**8**	**43**	**NA**	**6**	**5**	**7**	**6**	**3**	
18RB17252	*B. melitensis*	3	5	3	13	1	1	3	3	7	43	8	5	5	6	8	3	
18RB17253	*B. melitensis*	3	5	3	13	1	1	3	3	7	43	8	5	5	6	8	3	
18RB17254	*B. melitensis*	3	5	3	13	1	1	3	3	7	43	8	5	5	6	8	3	
18RB17258	*B. melitensis*	3	5	3	13	1	1	3	3	7	43	8	3	5	10	7	3	
**18RB17260**	***B. melitensis***	**3**	**5**	**3**	**13**	**1**	**1**	**3**	**3**	**7**	**48**	**NA**	**9**	**NA**	**8**	**9**	**3**	
**Reference**	***B. melitensis* 16M**	**3**	**4**	**2**	**13**	**4**	**2**	**3**	**3**	**5**	**36**	**6**	**2**	**5**	**7**	**3**	**6**	
