# Supplementary material for: Whole-Genome Sequencing for Tracing the Genetic Diversity of Brucella abortus and Brucella melitensis Isolated from Livestock in Egypt
Source: Pathogens. 2021 Jun 16;10(6):759. doi: 10.3390/pathogens10060759 (PMC8235727; doi:10.3390/pathogens10060759)
Supplement: Supplementary file 1 [file pathogens-10-00759-s001.zip › Table S4 SNP variant types of chromosomes 1 & 2 of B. abortus & B.pdf]

Table S4. SNP variant types for chromosome 1 (NZ_CP007681) and chromosome 2 (NZ_CP007680) of reference *B. abortus* BDW, and chromosomes 1 (NC_003317) and 2 (NC_003318) of reference *B. melitensis* 16M

SNP type	*B. abortus*				*B. melitensis*			
	NZ_CP007681		NZ_CP007680		NC_003317		NC_003318	
Missense variant	62	43.66%	60	52.63%	881	47.06%	534	48.06%
Splice region variant	0	0%	0	0%	3	0.16%	0	0
Start lost	0	0%	0	0%	1	0.05%	0	0
Stop gained	0	0%	2	1.75%	9	0.48%	5	0.45%
Stop lost	1	0.70%	0	0%	2	0.11%	0	0
Synonymous variant	43	30.28%	28	24.56%	548	29.27%	303	27.27%
Upstream gene	36	25.35%	24	21.05%	428	22.86%	260	23.40%
Downstream gene	0	0%	0	0%	0	0.00%	8	0.72%
Initiator codon variant	0	0%	0	0%	0	0.00%	1	0.09%
Total	142		144		1872		1111	

* Percentage of SNP type in relation to the total cgSNPs
