# Supplementary figures and images for: Whole-Genome Sequencing for Tracing the Genetic Diversity of Brucella abortus and Brucella melitensis Isolated from Livestock in Egypt
Source: Pathogens. 2021 Jun 16;10(6):759. doi: 10.3390/pathogens10060759 (PMC8235727; doi:10.3390/pathogens10060759)

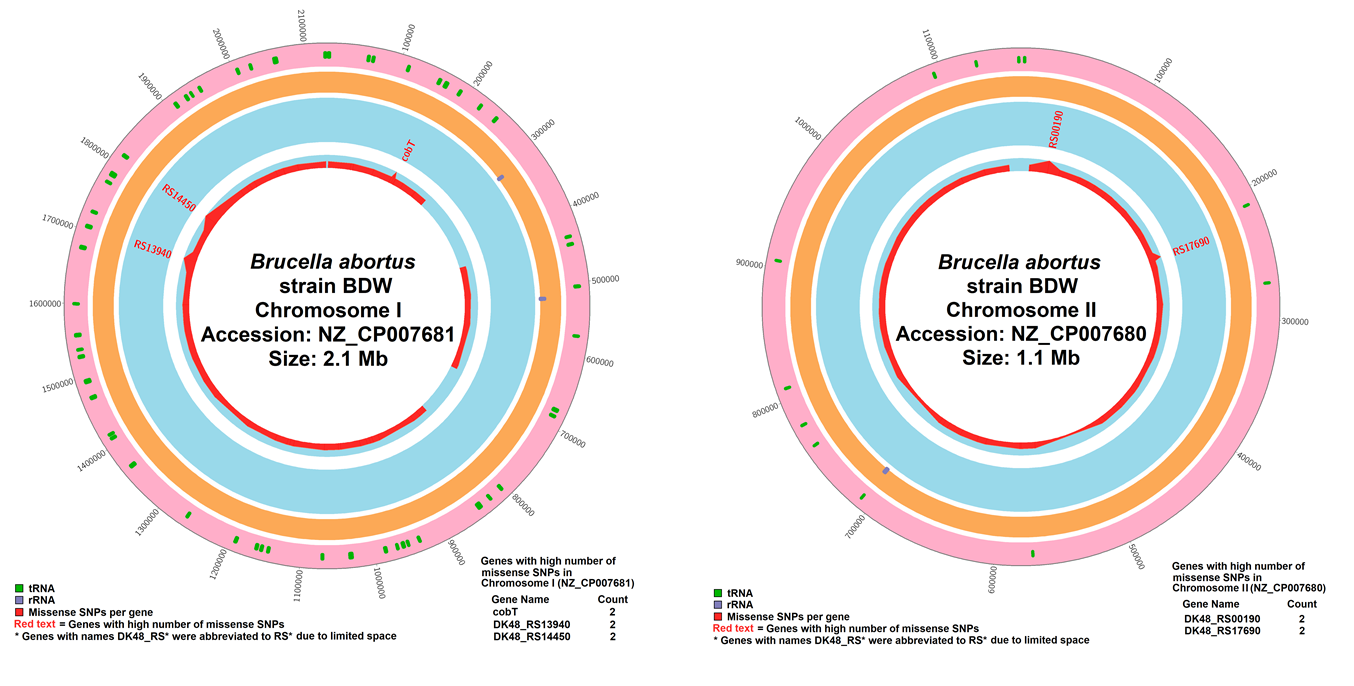

Supplement: Supplementary file 1 [file pathogens-10-00759-s001.zip › Fig. S1a & S1b. Circular presentation B abortus.png]

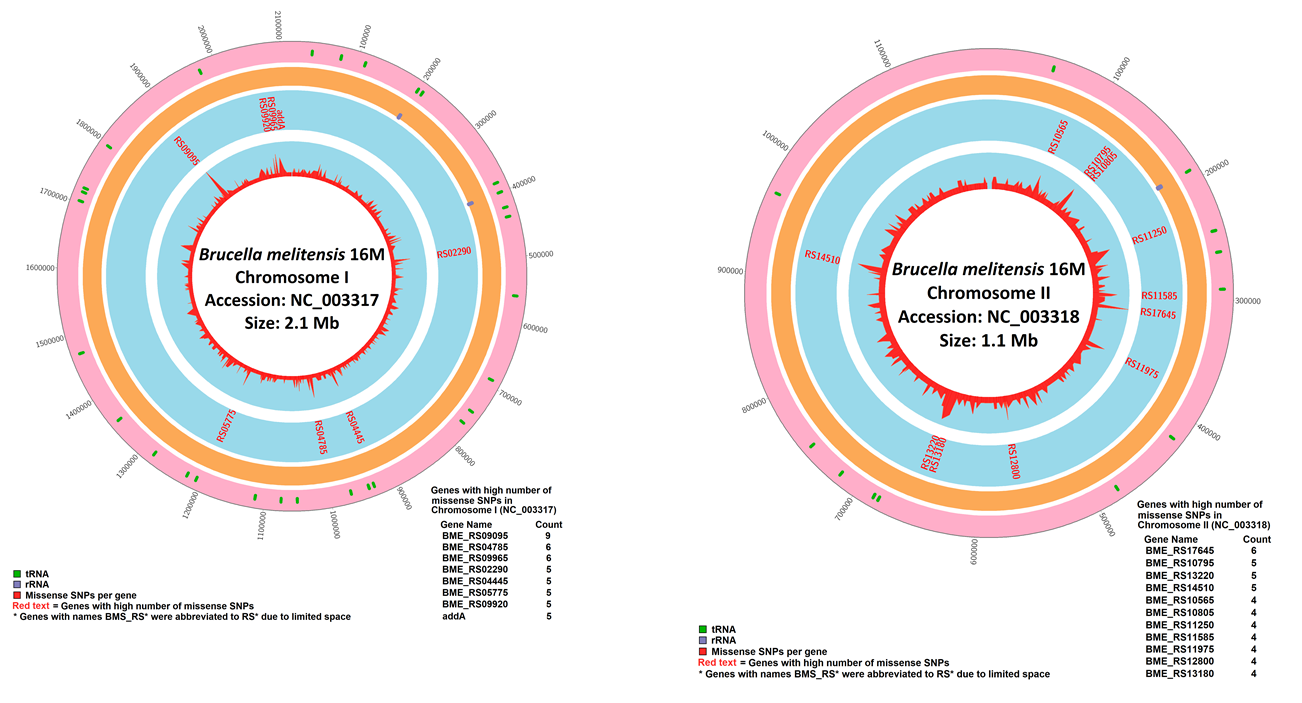

Supplement: Supplementary file 1 [file pathogens-10-00759-s001.zip › Fig. S1c & S1d. Circular presentation B melitensis.png]

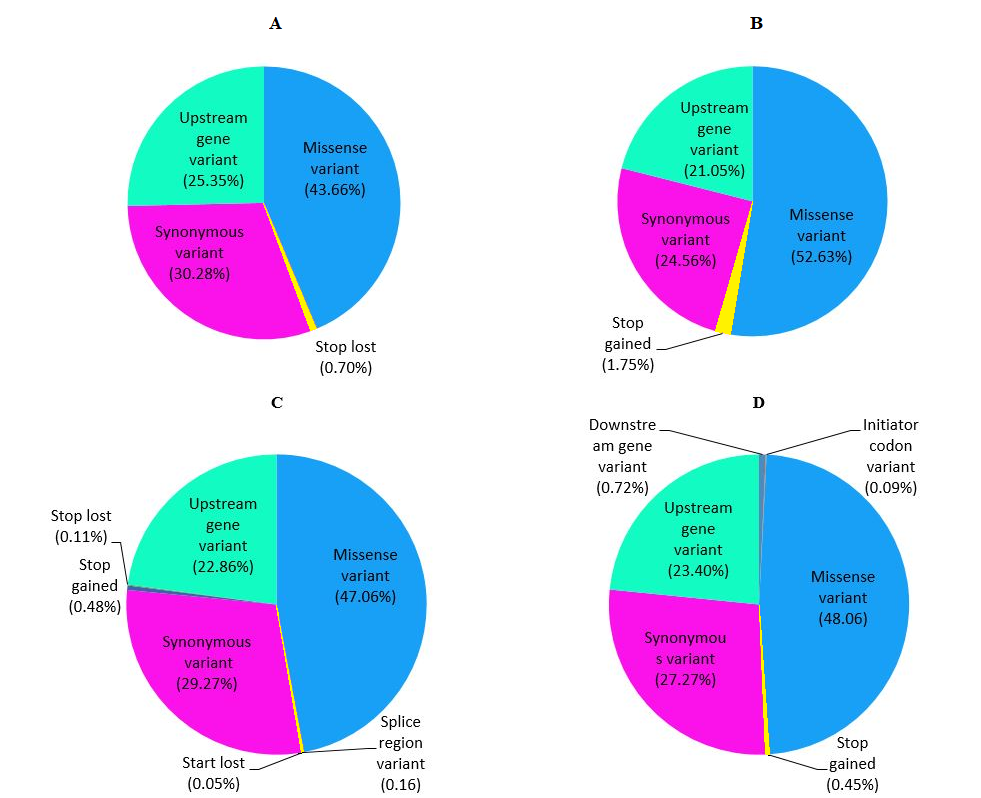

Supplement: Supplementary file 1 [file pathogens-10-00759-s001.zip › Fig. S2a to d. Percentages of SNP types for reference B. abortus BDW & reference B. melitensis 16M.tif]
